# Dexmedetomidine attenuates lung apoptosis induced by renal ischemia–reperfusion injury through α_2_AR/PI3K/Akt pathway

**DOI:** 10.1186/s12967-018-1455-1

**Published:** 2018-03-23

**Authors:** Juanjuan Li, Qian Chen, Xinhai He, Azeem Alam, Jiaolin Ning, Bin Yi, Kaizhi Lu, Jianteng Gu

**Affiliations:** 1 Department of Anesthesiology, Southwest Hospital, Third Military Medical University, 30 Gaotanyan Road, Chongqing, 400038 China; 2 Anaesthetics, Pain Medicine and Intensive Care, Department of Surgery and Cancer, Faculty of Medicine, Imperial College London, Chelsea & Westminster Campus, London, UK

**Keywords:** Renal ischemia–reperfusion, Acute lung injury, α_2_-Adrenoceptor, Akt pathway, Apoptosis

## Abstract

**Background:**

Acute lung injury caused by renal ischemia–reperfusion is one of the leading causes of acute kidney injury-related death. Dexmedetomidine, an α_2_-adrenergic agonist sedative, has been found to have protective effects against acute kidney injury and remote lung injury. We sought to determine whether dexmedetomidine can exert its anti-apoptotic effects in acute lung injury after acute kidney injury, in addition to its common anti-inflammatory effects, and to determine the underlying mechanisms.

**Methods:**

In vivo, acute kidney injury was induced by 60 min of kidney ischemia (bilateral occlusion of renal pedicles) followed by 24 h of reperfusion. Mice received dexmedetomidine (25 µg/kg, i.p.) in the absence or presence of α_2_-adrenergic antagonist atipamezole (250 µg/kg, i.p.) before IR. Histological assessment of the lung was conducted by HE staining and arterial blood gases were measured. Lung apoptosis was assessed by terminal deoxynucleotidyl transferase-mediated dUTP nick end-labeling assay. The expression of caspase 3 and p-Akt in lung tissue was detected by western blot. In vitro, C57BL/6J mice pulmonary microvascular endothelial cells were treated with serum from mice obtained following sham or IR. Dexmedetomidine was given before serum stimulation in cells, alone or with atipamezole or LY294002. Cell viability was assessed by CCK 8 assay. Cell apoptosis was examined by Hoechst staining and Annexin V-FITC/PI staining flow cytometry analysis. Mitochondrial membrane potential was measured by flow cytometry. The expression of p-Akt, caspase 3, Bcl-2 and Bax was measured by western blot.

**Results:**

In vivo, dexmedetomidine remarkably mitigated pathohistological changes and apoptosis and significantly increased p-Akt expression in the lung. In addition, dexmedetomidine also slightly improved oxygenation in mice after IR, which can be abolished by atipamezole. In vitro, dexmedetomidine significantly inhibited IR serum-induced loss of viability and apoptosis in PMVECs. Dexmedetomidine increased p-Akt in a time- and dose-dependent manner, and down-regulated the expression of caspase 3 and Bax and up-regulated the Bcl-2 expression in PMVECs. The changes of MMP were also improved by dexmedetomidine. Whilst these effects were abolished by Atipamezole or LY294002.

**Conclusion:**

Our results demonstrated that dexmedetomidine attenuates lung apoptosis induced by IR, at least in part, via α_2_AR/PI3K/Akt pathway.

## Background

Acute kidney injury (AKI) is a common and severe complication in critically ill patients, associated with high morbidity and mortality [1]. Renal ischemia–reperfusion (IR) is the major cause of AKI [2]. Ischemia AKI rarely occurs in isolation, and often contributes to multiorgan failure. Interestingly, pulmonary failure rather than kidney failure is one of the leading causes of AKI-related death [3, 4]. In addition, it is important to note that the high mortality associated with AKI cannot been appreciably improved by dialysis [5]. The mechanism of acute lung injury (ALI) caused by AKI is very complex, including volume overload resulting from kidney function loss and lung injury resulting from systemic inflammation [6]. Evidences suggest that lung endothelial apoptosis and lung inflammation mediate ALI after AKI [2, 6–8]. A few studies have found that ALI can be attenuated by various anti-inflammatory compounds [2, 9]. However, no pharmaceutical agents were found to possess an anti-apoptotic effect in ALI after AKI.

Apoptosis, or programmed cell death, plays an important role in a variety of disease states, and be regulated by several pathways [10]. Pulmonary microvascular endothelial cells (PMVECs) are a potential mediator between kidney and lung injury. Pro-inflammation and pro-apoptosis genes expression in PMVECs was significantly altered following AKI. Apoptosis of PMVECs results in disruption of the endothelial barrier and leads to pulmonary edema. Therefore, treatment focused on protecting PMVECs against injury may be an effective strategy in the therapeutic management of AKI induced-ALI.

Dexmedetomidine (Dex), a potent and highly selective α_2_-adrenergic agonist, exhibits sedative, analgesic, amnestic, and sympatholytic properties, and is widely used in critically ill and anesthetic patients. Our previous studies have found that Dex is able to protect against ALI after IR, predominantly due to its anti-inflammatory properties and its ability to ameliorate pulmonary microvascular hyper-permeability [11, 12]. The anti-apoptotic property of Dex has been demonstrated gradually in recent years [13–15]. In this study, we hypothesized the protective effect of Dex on ALI was involved in anti-apoptosis and investigated the potential mechanism.

## Methods

### Animal care

Experiments were performed on male C57BL/6J mice, 10-weeks old and weighing 25–30 g. The animals were obtained from the laboratory animal center of Third Military Medical. They were kept in a room with lights on from 8:00 to 20:00 at 25 ± 2 °C and a relative humidity of 55 ± 5%, and fed with a standard pellet diet and water ad libitum. The experiments were reviewed and approved by the Animal Care Committee of Third Military Medical University, Chong Qing, China.

### Surgical procedure

All mice were anesthetized with pentobarbital sodium (50 mg/kg i.p.) and placed on a heating pad to maintain the temperature at 36 ± 0.1 °C. Animals were randomly assigned to five groups (n = 6 per group): renal ischemia–reperfusion injury group (IR): bilateral renal pedicles were clamped for 60 min with a microvascular clamps. Then the clamps were gently removed; Sham group (Sham): laparotomy was performed without renal vessels occlusion. Dex group (Dex): Dex (Precedex 100 μg/mL Dex, Orion Pharma, Espoo, Finland) was intraperitoneally injected at 25 μg/kg without surgery. Pre-treatment with Dex group (Dex + IR): 25 μg/kg Dex 15 min prior to ischemia was administered. Combination of the α_2_-adrenergic antagonist atipamezole (Atip + Dex + IR): atipamezole (Sigma-Aldrich, St. Louis, MO, USA) 250 μg/kg was administered 10 min prior to Dex pre-treatment. All animals were administered 0.5 mL of sterile saline intraperitoneally, and the incision closed in two layers with a 4–0 silk. At the end of 24 h reperfusion, the mice were euthanized by exsanguination under pentobarbital sodium anesthesia, and tissues were collected for analysis. Blood samples from abdominal aorta were obtained from Sham and IR group animal, and were centrifuged (3000*g*, 4 °C for 20 min) to obtain serum, which was then stored at − 80 °C.

### Hematoxylin and eosin staining

After paraffin embedding, lung tissues were sectioned into 5-μm thick sections. Then the sections were stained with hematoxylin/eosin and examined under a light microscope. The degree of lung injury was scored on a scale from 0 to 3 using a previously described scoring system: Grade 0, normal pulmonary appearance; Grade 1, mild moderate interstitial congestion and neutrophil leukocyte infiltrations; Grade 2, perivascular edema formation, partial leukocyte infiltration, moderate neutrophil leukocyte infiltration; Grade 3, severe destruction of the lung architecture and massive neutrophil leukocyte infiltration.

### Terminal deoxynucleotidyl transferase transfer-mediated dUTP nick end-labeling staining (TUNEL)

TUNEL assay was conducted to evaluate apoptosis using an In Situ Cell Death Detection Kit, POD (Roche, Germany) according to the manufacturer’s instruction. Lung tissues were fixed with 4% paraformaldehyde and embedded in paraffin. Then the tissues were sectioned at 5 μm for TUNEL staining. After deparaffinization and rehydration, the sections were applied with 10 μg/mL protease K for 15 min. Fresh TUNEL reaction mixture was added on the samples which were incubated for 60 min at 37 °C in the dark. After washing, the cell nucleuses were stained with 0.1 μg/mL DAPI (beyotime, China). The samples were analyzed in a drop of PBS under a fluorescence microscope (Olympus, Japan). The number of TUNEL-positive cells per high-power field was calculated and analyzed in a blinded manner by observing eight random visual fields per animal under a magnification of 200×.

### Arterial blood gas analysis

Arterial blood samples were obtained for blood gas analysis. A 0.5-mL sample of arterial blood was drawn from the abdominal aorta, and pH, partial pressure of oxygen (PaO_2_) and partial pressure of carbon dioxide (PaCO_2_) were measured at the end of the reperfusion period with a blood gas analyzer (Beckman Coulter, Inc., USA).

### Cell culture

C57BL/6J mice PMVECs were purchased from the Cell Biologics Inc. (Chicago, USA). The cells were cultured in M-1168 medium (Cell Biologics, Chicago, USA) at 37 °C and in a humidified atmosphere of 5% CO_2_. Cells between passage 3 and 6 were used in the subsequent experiments.

### Cell viability analysis

Cell viability was monitored using a CCK-8 assay (Dojindo, Japan) according to the manufacturer’s protocol. PMVECs were plated at a density of 5 × 10^3^ cells/well in 96-well plates. After overnight culture, the culture medium was removed, after which the cells were incubated with different doses of Dex (0.1, 1, 10 μM) or different concentrations of IR serum (10, 15, 20, 25%) for 24 h, or pre-incubated with different concentrations of Dex for 1 h before exposure to 15% IR serum for 24 h. Meanwhile, cells incubated with 15% sham serum for 24 h was used as the control group. After chemical stimulation, the supernatant was removed, CCK-8 reagent (10 μL) was added to each well of a 96-well plate containing 100 μL culture medium and the plate was incubated for 3 h at 37 °C. Cell viability was evaluated by absorbance measurements at 450 nm.

### Hoechst 33258 staining

PMVECs were cultured in 6-well plates at a density of 4 × 10^4^ cells per well overnight, after which cells were exposed to various concentrations of Dex for 1 h, followed by exposure to 15% IR serum for 24 h. After these treatments, PMVECs were fixed in paraformaldehyde (4%) for 10 min, washed with PBS buffer, and then stained with the Hoechst 33258 (Beyotime Biotechnology Company, China) for 5 min. Photomicrographs were captured under an inverted fluorescence microscope Nikon EclipseTE300 inverted microscope (Nikon, Tokyo, Japan) at an excitation wavelength of 350 nm excitation and an emission wavelength of 460 nm Annexin V-FITC/PI double staining study using flow cytometry.

### Cell apoptosis analysis

Cells apoptosis was examined by double staining with Annexin V-FITC Apoptosis Detection Kit (Bestbio, China). PMVECs were seeded in 6-well plates at a density of 1 × 10^5^ cells/mL, and incubated overnight. Then cells were treated with different concentrations of Dex for 1 h prior to exposure to 15% IR serum for 24 h. After being washed twice with cold PBS, cells were suspended in 400 μL binding buffer at a concentration of 1 × 10^6^ cells/mL, after which cells were incubated in 5 μL Annexin V-FITC for 10 min and 10 μL PI for 15 min at 4 °C in the dark. Cells were subsequently analyzed using flow cytometry (ACEA, Biosciences Inc., China).

### Measurement of mitochondrial membrane potential (MMP)

A mitochondrial membrane potential assay kit with JC-1 (Beyotime, China) was used to detect the mitochondrial membrane potential. According to the manufacturer’s protocol, PMVECs were seeded in 6-well plates at a density of 1 × 10^5^ cells/mL, and incubated overnight. Cells incubated with 15% Sham serum or IR serum for 24 h were used as the control group or IR group, respectively. The Dex + IR group was pretreated with 0.1 μM Dex 1 h before the IR group. In the Atip + Dex + IR group, 1 μM atipamezole was administered 10 min prior to dexmedetomidine pre-treatment. The LY294002 + Dex + IR group was treated with 20 μM LY294002 for 30 min before Dex treatment. After these treatment, cells were incubated with JC-1 working solution for 20 min at 37 °C in the dark after treatment, and then washed twice with ice-cold JC-1 buffer solution before analysis with flow cytometry.

### Western blot

The lung tissues and cells were harvested at the end of the experiment. Protein was extracted from tissue or cells in a lysis buffer. After estimate protein concentrations with a bicinchoninic acid protein assay kit, equal amounts of protein (40 μg) from each sample were separated and electrotransferred onto a PVDF membranes. The membranes were then blocked in 5% BSA and incubated overnight at 4 °C with primary antibodies. After washing three times in TBST, the membranes were then incubated with HRP-coupled secondary antibodies. The antibodies are as follows: rabbit monoclonal anti-cleaved caspase-3 antibody (1:1000, Cell Signaling Technology, USA), rabbit monoclonal anti-Bcl-2 antibody (1:1000, Cell Signaling Technology, USA), rabbit monoclonal anti-Bax antibody (1:1000, Cell Signaling Technology, USA), rabbit monoclonal anti-phospho-Akt antibody (1:1000, Ser473, Cell Signaling Technology, USA), anti-rabbit IgG, HRP- linked antibody (1:2500, Cell Signaling Technology, USA), rabbit polyclonal anti-β-actin antibody (1:2000, Solarbio, China). Bands were detected using standard ECL (Millipore, USA) and quantified by densitometric analysis using Image J software (Version 1.44p, National Institutes of Health, Bethesda, MD, USA).

### Statistical analysis

Data were expressed as mean ± SD. All statistical analyses were performed using GraphPad Prism 5.01. One-way analysis of variance followed by post hoc Newman–Keuls test were used for comparison, otherwise, unpaired two tailed Student test was used wherever appropriate. A p value less than 0.05 was considered to be of statistical significance.

## Results

### Dex improved the pathological structure and arterial blood gas changes of the lungs following renal IR in mice

**Fig. 1:**
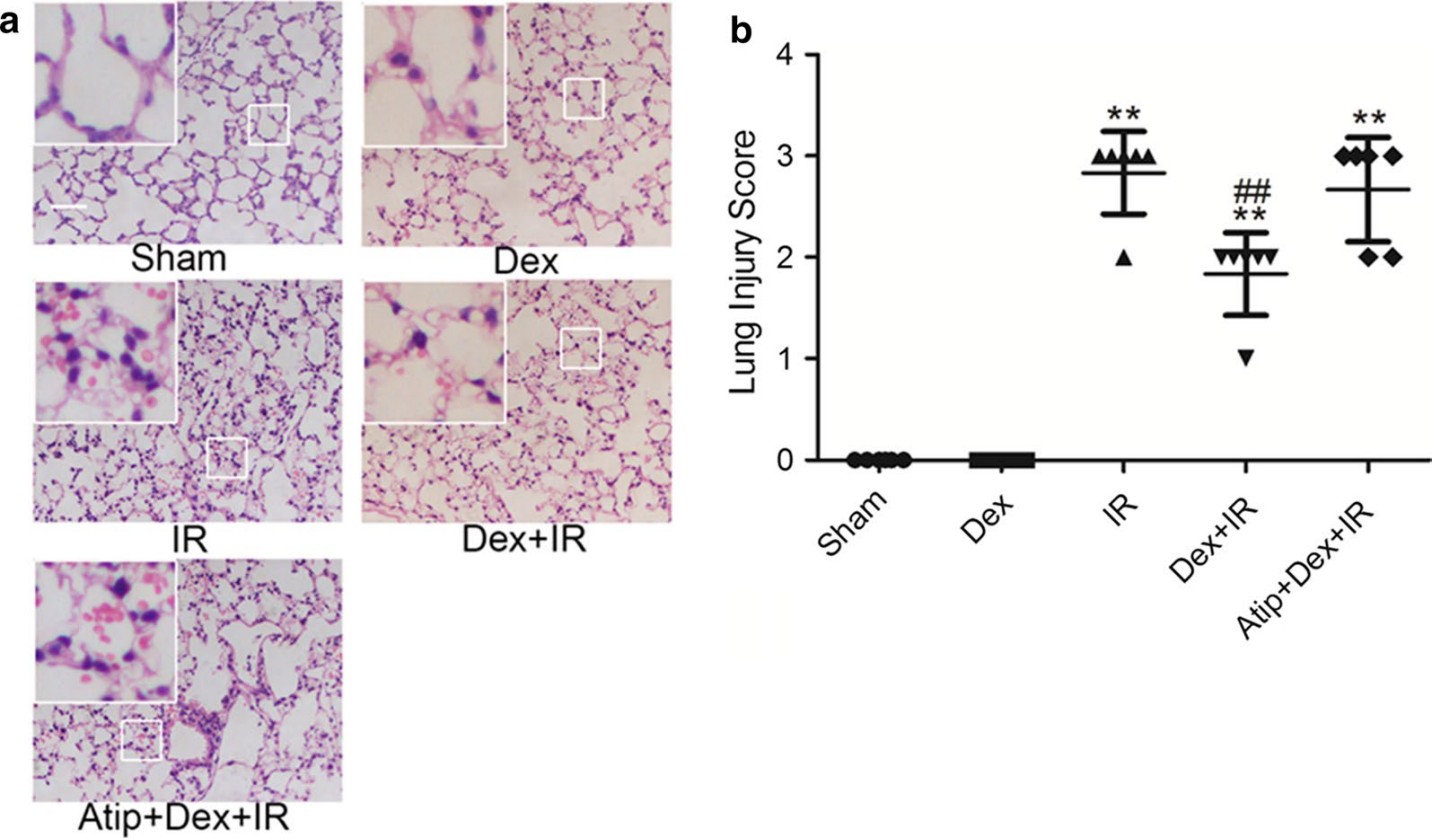
Dexmedetomidine preserved lung architecture in renal IR—induced lung injury. C57B/6J mice were pre-treated with dexmedetomidine (Dex) alone or in combination with α_2_-adrenoceptor antagonist atipamezole (Atip) followed by clamping of the bilateral renal pedicle for 60 min and reperfusion for 24 h. Sham animals were used as control. Representative photomicrographs of pulmonary histology (**a**) and lung injury scores (**b**) were evaluated under different conditions. Bar represents a length of 500 μm on histology. Data are shown as mean ± SD. n = 6 per group, **p < 0.01 versus sham group, ^##^p < 0.01 versus IR group

To determine the effect of Dex on lung injury after IR, HE staining for lung histology was evaluated (Fig. 1a). The alveoli in the sham or Dex group was integrated and there was no significant exudation in the alveoli. Notably, disordered alveolar structure was found in the IR group, with a significant pulmonary interstitial edema, and a large number of red blood cells and inflammatory cells in the alveolar cavity. However, pre-treatment with Dex (Dex + IR) alleviated lung injury, as evidence by intact alveolar structure, an improvement in interstitium edema, and a reduction in red cells and inflammatory cells alveolar infiltration. The protective effects of Dex were reversed by α_2_-adrenoceptor antagonist atipamezole (Atip + Dex + IR). All of these changes were corroborated by histological scores (Fig. 1b).

Mice arterial blood gas analysis was performed to further assess the lung injury (Table 1). The arterial blood pH value in the IR group decreased after the experiment compared with sham or Dex group. Acidosis can be improved significantly with Dex pre-treatment (p < 0.05). However, atipamezole abolished this effect. The arterial partial pressure of oxygen and arterial blood CO_2_ decreased in IR group compared to the sham or Dex group (p < 0.01), which was improved slightly by pre-treatment with Dex, however there was no statistical significance.

**Table 1:** Results of arterial blood gas analyses in the five groups

Group	pH	PaO_2_ (mmHg)	PaCO_2_ (mmHg)
Sham	7.28 ± 0.02	117.50 ± 5.68	38.00 ± 2.61
Dex	7.29 ± 0.02	117.83 ± 2.14	38.67 ± 2.16
IR	7.03 ± 0.05**	93.67 ± 5.01**	29.83 ± 1.17**
Dex + IR	7.09 ± 0.01**^,#^	98.83 ± 1.47**	31.33 ± 1.03**
Atip + Dex + IR	7.00 ± 0.07**	92.17 ± 6.18**	29.00 ± 1.41**

C57B/6J mice were pre-treated with dexmedetomidine (Dex) alone or in combination with α_2_-adrenoceptor antagonist atipamezole (Atip) followed by clamping of the bilateral renal pedicle for 60 min and reperfusion for 24 h. Sham animals were used as control. Data are shown as mean ± SD. n = 6 per group, ** p < 0.01 versus sham group, ^#^ p < 0.01 versus IR group

### Dex ameliorated lung apoptosis induced by IR in mice

**Fig. 2:**
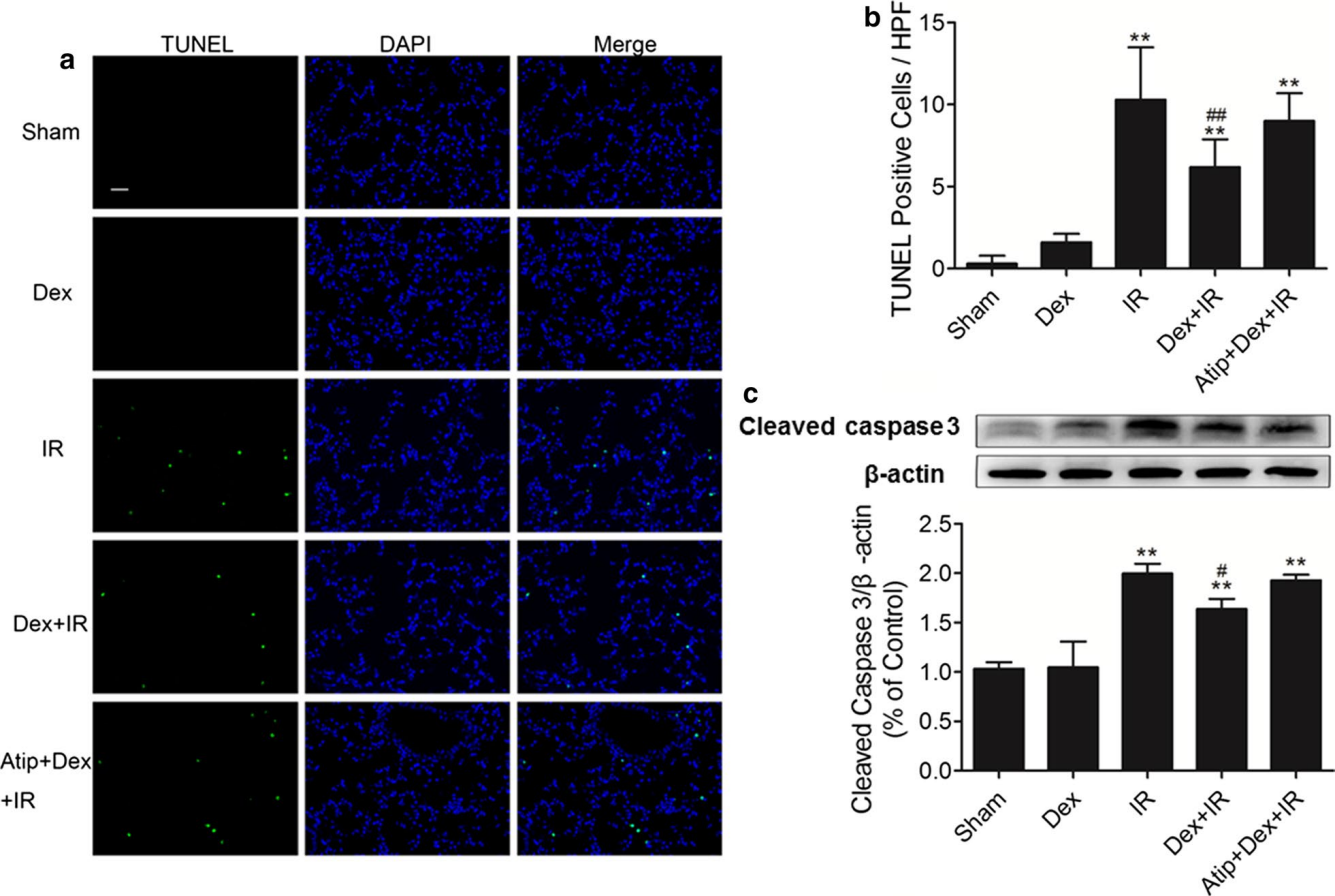
Dexmedetomidine inhibited lung apoptosis induced by IR in mice. C57B/6J mice were pre-treated with dexmedetomidine (Dex) alone or in combination with α2-adrenoceptor antagonist atipamezole (Atip) followed by clamping of the bilateral renal pedicle for 60 min and reperfusion for 24 h. **a** Apoptosis in the lungs was assessed by a TUNEL assay. **b** Quantitative analysis of apoptotic cells in kidneys. **c** Representative western blotting results and quantitative analysis of cleaved caspase-3 in lung tissues. The scale bars in all images represent 50 μm. All data are expressed as the mean ± SD (n = 6). **p < 0.01 versus sham group, ^#^p < 0.05 versus IR group. HPF means high-power field

The terminal deoxynucleotidyl transferase-mediated dUTP nick end-labeling (TUNEL) assay was employed to determine whether Dex attenuates lung apoptosis after IR in mice (Fig. 2a, b). The apoptotic cells of lung tissue in IR group were obviously increased compared with the Sham or Dex group (p < 0.01),and were dramatically decreased in the Dex + IR group (p < 0.01). However, the effect of Dex was abolished by atipamezole. As a major executive caspase in apoptosis, activated caspase 3 was measured by western blot in different groups (Fig. 2c). We found that the expression of cleaved caspase 3 in IR group was increased significantly compared with that of the Sham group or the Dex group (p < 0.01). Furthermore, the Dex + IR group had a decreased level of cleaved caspase 3 (p < 0.05). By contrast, the effect of Dex was abrogated in the Atip + Dex + IR group. All of these findings suggest that Dex protects against lung apoptosis induced by IR, which might be via α_2_ adrenoceptor-mediated mechanism.

### Dex improved decreased cell viability induced by IR mice serum

**Fig. 3:**
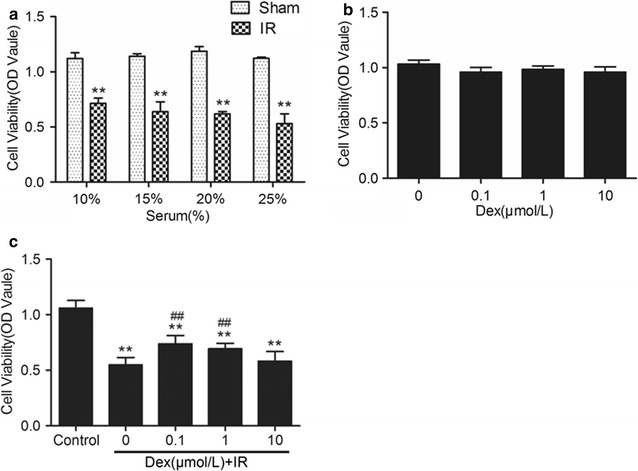
Dexmedetomidine inhibited mice IR serum-induced loss of viability in PMVECs. **a** Cell viability of PMVECs treated with different concentrations (10, 15, 20, 25%) of sham or IR serum. **b** Cell viability of PMVECs treated with different concentrations (0.1, 1, 10 μM) of Dexmedetomidine. **c** Cells were treated with 0.1–10 μM DEX 1 h before receiving 15% IR serum stimulation. The 15% sham serum stimulated group were used as control. **p < 0.01 versus Control group, ^##^p < 0.01 versus IR group

In order to assess the effect of IR mice serum and Dex on PMVECs, cell viability represented by the absorbance value was measured using CCK8 assay. As illustrated in Fig. 3a, compared with the Sham group, the IR group cells’ viability significantly decreased (p < 0.01) at the serum concentration of 10, 15, 20 and 25%, respectively. As seen in Fig. 3b, exposure to 0.1–10 μM Dex for 24 h did not affect cell viability. The cells were then treated with 0.1–10 μM Dex 1 h before receiving 15% IR serum stimulation, as seen in Fig. 3c, 0.1 and 1 μM Dex significantly attenuated (p < 0.01) the cell viability loss induced by IR serum, whilst 10 μM Dex had no effect.

### Dex inhibited IR mice serum-induced PMVECs apoptosis

**Fig. 4:**
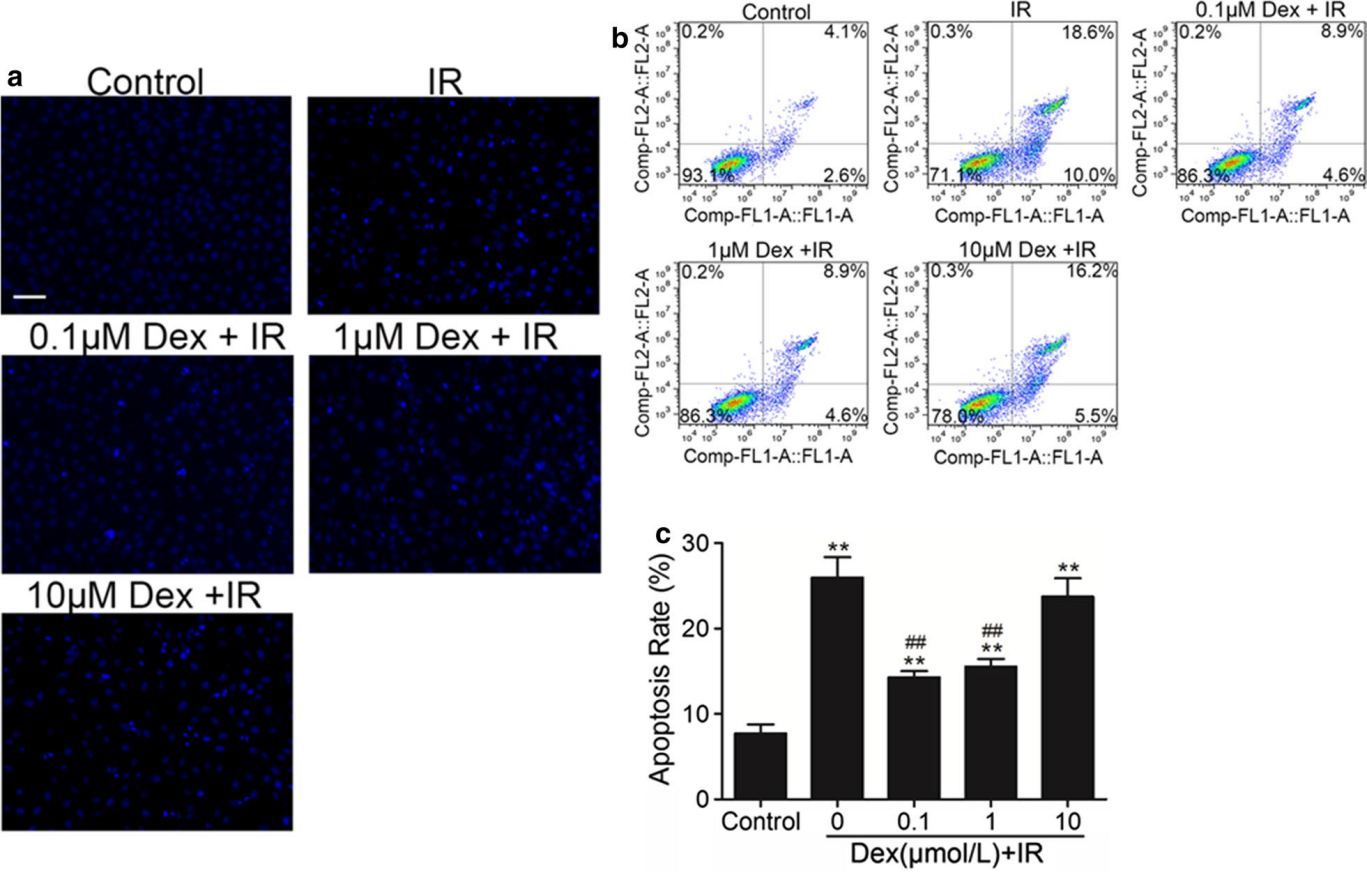
Dexmedetomidine inhibits IR serum-Induced apoptosis of PMVECs. Cells were treated with 0.1–10 μM DEX 30 min before receiving 15% IR serum stimulation. **a** Hoechst 33258 staining in PMVECs. **b** Cell apoptosis measured by flow cytometry. **c** The percentage of apoptotic cells. The 15% sham serum stimulated group was used as control. Bar represents a length of 50 μm. Data are shown as mean ± SD. **p < 0.01 versus sham group, ^##^p < 0.01 versus IR group

Hoechst 33258 staining was used to investigate the apoptosis in PMVECs (Fig. 4a). This dye is shiny blue when in contact with apoptotic cells, whilst it is light blue in normal cells. Cells treated with 15% IR serum exhibited increased apoptosis compared with control group, whereas that was decreased by Dex at 0.1 or 1 μM. However, 10 μM DEX showed no protective effect.

To further confirm the anti-apoptotic effect of Dex, we detected cell apoptosis by Annexin V-FITC/PI double staining using flow cytometry. As shown in Fig. 4b, c, compared with the control group, the apoptotic rates was greatly increased in the IR group (p < 0.01). Cells pre-treated with 0.1 or 1 μM Dex had a reduced apoptotic rate (p < 0.01). On the contrary, 10 μM Dex didn’t protect PMVECs against apoptosis. All of these findings were consistent with the results of dyeing. Based on these results, 0.1 μM DEX was selected for subsequent studies.

### Dexmedetomidine promotes Akt phosphorylation in vivo and vitro

**Fig. 5:**
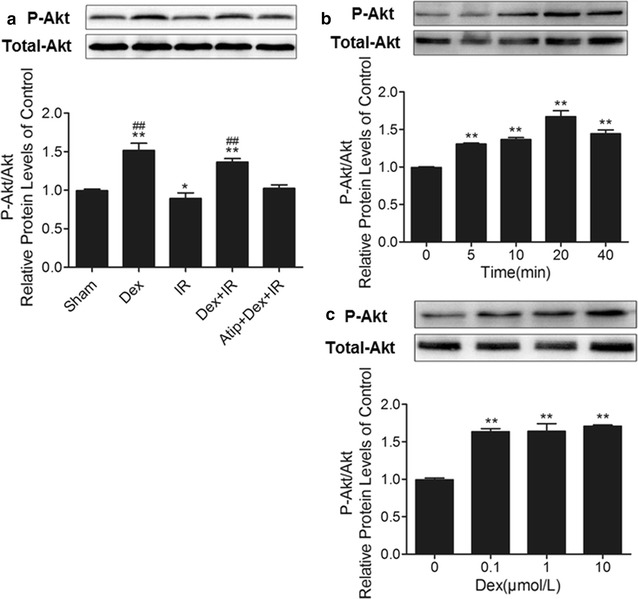
Effects of dexmedetomidine on the expression of p-Akt in vivo and vitro. **a** Representative western blotting results and quantitative analysis of p-Akt in lung tissues. **b** Effects of dexmedetomidine (0.1 μM) on the expression of p-Akt in PMVECs in different time (5, 10, 20, 40 min). **c** Effects of different concentrations (0.1, 1, 10 μM) of dexmedetomidine on the expression of p-Akt in PMVECs. **p < 0.01 versus control group, ^#^p < 0.05 versus IR group, ^##^p < 0.01 versus IR group

In vivo, lung tissue was harvested after 24 h reperfusion and p-Akt was measured. As shown in Fig. 5a, Dex up-regulated the expression of p-Akt in the Dex + IR group, while the IR group exhibited a decreased expression of p-Akt compared to the sham group (p < 0.05). However, this effect was abolished in the Atip + Dex + IR group. In vitro, PMVECs were incubated with 0.1 μM Dex for 5, 10, 20, and 40 min. As shown in Fig. 5b, exposure of PMVECs to 0.1 μM Dex for 20 min significantly induced Akt phosphorylation (p < 0.01). At last, PMVECs were incubated with 0.1, 1 and 10 µM Dex for 20 min. As shown in Fig. 5c, Dex led to a linear increase in Akt phosphorylation.

### The α2AR/PI3K/Akt signaling pathway involved in the PMVECs protection of Dex

To investigate whether the anti-apoptotic effect of Dex was associated with the α_2_AR/PI3K/Akt pathway, the α_2_AR antagonist atipamezole and the PI3K inhibitor LY294002 were pre-treated with Dex. Compared to the control group and the Dex group, cleaved caspase 3 expression significantly increased in IR group (p < 0.01), whereas, pre-treatment with Dex strongly attenuated it. However, in the Atip + Dex + IR or the LY294002 + Dex + IR group, Dex-induced reduction in caspase 3 expression became blunted, suggesting that the protective effect of Dex may involve the α_2_AR/PI3K/Akt pathway.

**Fig. 6:**
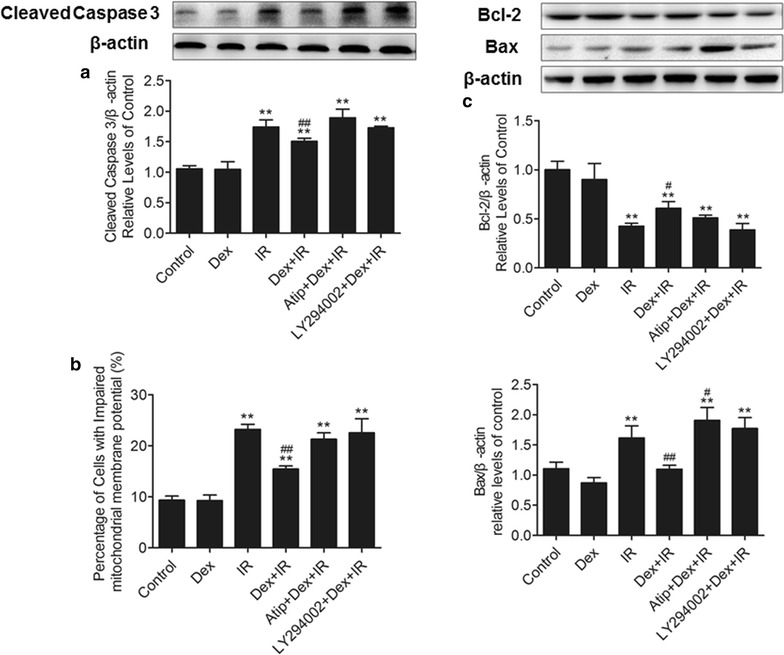
The α_2_AR/PI3K/Akt signaling pathway may be involved in the PMVECs protection of Dex. PMVECs were pretreated with Atipamezole or the PI3K inhibitor LY-294002 30 min before 0.1 μM dexmedetomidine treatment for 20 min, followed by 15% IR serum stimuli for 24 h. The 15% sham serum stimulated group was used as the control. **a** The cleaved caspase in PMVECs was detected by western blot. **b** Cells were stained with JC-1 and mitochondrial membrane potential was determined by flow cytometry. **c** The expression of Bcl-2 and Bax in PMVECs. **p < 0.01 versus control group, ^#^p < 0.05 versus IR group, ^##^p < 0.01 versus IR group

In order to investigate whether the anti-apoptotic effect of Dex may be related to the mitochondrial pathway, mitochondrial membrane potential (MMP) was detected by flow cytometry (Fig. 6b). We found that impaired MMP in PMVECs induced by IR serum was alleviated by Dex. Consistently, atipamezole or LY294002 could reverse the effect.

We next investigated the influence of α_2_AR/PI3K/Akt pathway on Bcl-2 and Bax expression; these proteins belong to Bcl-2 protein family that regulates mitochondrial apoptosis. As presented in Fig. 6c, the expression of Bcl-2 was down-regulated and Bax was up-regulated in PMVECs exposed to IR serum for 24 h. These effects were partially reversed by the pretreatment with 0.1 μM DEX. Atipamezole or LY294002 can reversed the effects of Dex on the expression of Bcl-2 and Bax.

## Discussion

AKI frequently leads to ALI in critically ill patients. The mortality of combined AKI and ALI is extremely high, and may approach 80% [16]. The underlying pathophysiological mechanisms are extremely complex. Within 6 h of renal injury, lung disease and increased pulmonary vascular permeability are intrinsically associated with lung inflammation [3]. Multiple lines of evidence have found that apoptosis contributes to lung injury at 24 h [7, 8, 17]. In recent years, some anti-inflammatory agents, including α-MSH [9] and IL-6 inhibitors [18], have been shown to protect against lung injury after AKI. However, no anti-apoptotic agents have been found to protect against ALI induced by AKI. Our previous study found that Dex could attenuate lung inflammation and the pulmonary microvascular hyper-permeability. In the present study, we demonstrated that Dex decreased lung injury and apoptosis after AKI in vivo. Furthermore, in order to investigate the underlying protective mechanisms at a cellular level, we established an in vitro model. Similarly, Dex protects PMVECs against apoptosis associated with IR injury, and the effect may be associated with α_2_AR/PI3K/Akt pathway.

ALI after IR is characterized by increased vascular permeability, interstitial edema, alveolar hemorrhage, and red blood cells sludging [19]. HE staining in our study also exhibited the increase injury level in IR group mice. Pre-treatment with Dex dramatically attenuated these pathological changes in the lung tissue of mice, which is consistent with our previous study [11]. Hypoxemia is an important clinical feature in ALI patients. Therefore, we detect the mice arterial blood gas. Acidosis and impaired oxygenation was observed in the IR group and Dex significantly alleviated the acidosis and slightly improved oxygenation demonstrated Dex might be able to reduce lung injury.

Previous evidence showed that pulmonary apoptosis after IR was caspase-dependent [17]. Caspase 3 is the major executive caspase in apoptosis [20]. Cleavage of caspase 3 results in its activation, thus facilitating its pro-apoptotic effects [21]. In our study, mice suffering from IR showed significant increased TUNEL-positive cells and cleaved caspase 3 expression in the lung tissue. However, lung apoptosis was improved in the Dex + IR group, which was also abolished by atipamezole. These findings suggest that the anti-apoptotic effect of Dex is at least partly via α_2_AR.

Recent evidence suggests that pulmonary apoptosis following IR is dominated by vascular endothelial cell apoptosis, rather than epithelial apoptosis [7, 22]. Therefore, PMVECs were chosen for the experiments. PMVECs exposed to IR serum exhibited a significant decrease in cell viability compared to those exposed to sham serum. However, 0.1 and 1 μM can significantly attenuated the loss in cell viability when cells were exposed to 15% IR serum. The effect of Dex reduce PMVECs apoptosis was confirmed by Hoechst 33258 nuclear staining and Annexin V-FITC/PI staining flow cytometry analysis. These results demonstrated that low concentrations of Dex (0.1–1 μM) significantly decreased the apoptosis of PMVECs resulting from 15% IR serum insult. However, a high concentration of Dex (10 μM) does not confer further protective benefit and can result in deleterious effects. In our previous study, we also found that 10 μM Dex lost its ability to reduce hyper-permeability of PMVECs monolayer. Another study demonstrated that high cumulative dose and concentration of Dex induce neuroapoptosis, in vivo and in vitro. But this effect could be reversed by co-administration of α_1_-adrenergic receptor blocker [23]. These findings indicate that Dex cytotoxicity is associated with off-target α_2_-adrenergic activation.

The PI3K/Akt signaling pathway plays a central role in cell growth, differentiation and apoptosis. In vivo, pretreatment with Dex significantly inhibited the IR induced decrease in p-Akt expression, which was weakened by the administration of atipamezole. These results suggest that Dex-mediated Akt phosphorylation may be via the α_2_AR signaling pathway. In vitro, Dex alone induced phosphorylation of Akt in a dose- and time-dependent manner. LY294002 was used to block the activation of PI3K/Akt pathway. The expression of cleaved caspase 3 demonstrated that both atipamezole and LY294002 could abolish the anti-apoptotic effect of Dex. Overall, Dex inhibits lung or PMVECs apoptosis, at least partly, via the α_2_AR/PI3K/Akt pathway.

Cell apoptosis is predominantly initiated by two distinct signaling pathways. One is the extrinsic (death receptor-dependent) pathway, and the other is the intrinsic (mitochondria-dependent) pathway. The extrinsic pathway is triggered when cell-surface death receptors, such as Fas, are bound by their ligands. The intrinsic pathway is mediated by the loss of integrity of the mitochondrial outer membrane, which causes the release of mitochondrial proteins such as cytochrome c from the mitochondrial into the cytosol. Subsequently, caspase 9 is activated, which in turn induces the activation of apoptotic caspases, such as caspase 3. The mitochondrial pathway is controlled by the Bcl2 protein family, which can be either pro-apoptotic (e.g. Bid, Bim, Bad, and Noxa) or anti-apoptotic (e.g. Bcl2, Bcl-xL, and Mcl1). PMVECs treated with IR serum exhibited MMP collapse, up-regulation of Bax and down regulation of Bcl-2, all of which can be improved by Dex. However, the effect can be abolished by atipamezole or LY294002. These results suggest that Dex may protect PMVECs from apoptosis by mediating mitochondrial signaling, which may involve modulation of the α_2_AR/PI3K/Akt pathway.

The current study is not without limitations. We used the serum from mice with renal ischemia–reperfusion injury to stimulate the cells, however, this in vitro model could not fully simulate the complex pathophysiological environment in the in vivo setting. Both serum inflammatory mediators and circulating leukocytes have been shown to contribute to remote lung injury after AKI [24], and the impact of infiltrating leukocytes on the apoptosis of PMVECs has not been explored in the present study and certainly warrants further investigation. However, our study demonstrated that serum from mice with renal ischemia–reperfusion injury contained injurious mediators, including pro-inflammatory cytokines and DAMP molecules, which were sufficient to damage lung cells. This indicates that circulating inflammatory mediators could serve as an essential target for developing therapeutic strategy against remote vital organ injury associated with renal IR injury.

## Conclusions

In summary, we found that Dex exerts anti-apoptotic effects against ALI induced by AKI, thus demonstrating a novel protective mechanism against remote lung injury, and indicating that Dex may be a promising therapeutic avenue in remote organ cross-talk.

## Abbreviations

ALI: acute lung injury
IR: ischemia–reperfusion
PMVECs: pulmonary microvascular endothelial cells
Dex: dexmedetomidine
TUNEL: terminal deoxynucleotidyl transferase transfer-mediated dUTP nick end-labeling staining
PaO_2_: partial pressure of oxygen
PaCO_2_: partial pressure of carbon dioxide
MMP: mitochondrial membrane potential
